# Sensor Fusion for Recognition of Activities of Daily Living

**DOI:** 10.3390/s18114029

**Published:** 2018-11-19

**Authors:** Jiaxuan Wu, Yunfei Feng, Peng Sun

**Affiliations:** 1 Department of Computer Science, Iowa State University, Ames, IA 50010, USA; jiaxuanw@iastate.edu (J.W.); psun@iastate.edu (P.S.); 2 College of Computer Science and Engineering, Northeastern University, Shenyang 110000, China

**Keywords:** activity of daily living, time-series, sensor fusion, smartphone, machine learning, big data

## Abstract

Activity of daily living (ADL) is a significant predictor of the independence and functional capabilities of an individual. Measurements of ADLs help to indicate one’s health status and capabilities of quality living. Recently, the most common ways to capture ADL data are far from automation, including a costly 24/7 observation by a designated caregiver, self-reporting by the user laboriously, or filling out a written ADL survey. Fortunately, ubiquitous sensors exist in our surroundings and on electronic devices in the Internet of Things (IoT) era. We proposed the *ADL Recognition System* that utilizes the sensor data from a single point of contact, such as smartphones, and conducts time-series sensor fusion processing. Raw data is collected from the *ADL Recorder App* constantly running on a user’s smartphone with multiple embedded sensors, including the microphone, Wi-Fi scan module, heading orientation of the device, light proximity, step detector, accelerometer, gyroscope, magnetometer, etc. Key technologies in this research cover audio processing, Wi-Fi indoor positioning, proximity sensing localization, and time-series sensor data fusion. By merging the information of multiple sensors, with a time-series error correction technique, the *ADL Recognition System* is able to accurately profile a person’s ADLs and discover his life patterns. This paper is particularly concerned with the care for the older adults who live independently.

## 1. Introduction

In the era of Internet of Things (IoT), activity recognition has mostly been studied in supervised laboratory settings. Many such studies are concerned with the quality of life of older adults who live independently in places such as at home. Many kinds of sensors widely exist in our daily environments, including in a house, in a car, in an airplane or when using a smartphone. To date, many researchers have utilized sensors to detect and recognize people’s activities of daily living. Activities of daily living (ADLs), instrumental ADLs (iADLs) [1] and enhanced ADLs (eADLs) [2] include a wide spectrum of tasks to maintain one’s desired living conditions such as eating, sleeping, using a toilet, bathing or showering, dressing, using a telephone, shopping, preparing meals, housekeeping, doing laundry, managing medications, etc. Much valuable information could be mined from the ADL data to analyze the types of difficulties encountered in everyday activities and predict trends.

For older adults who have chronic diseases or live in rural areas without sufficient healthcare, doing physical activities can have a positive impact on their well-being, not only at an individual level but also for the society as a whole. General measures of health status, such as diagnoses or medical conditions, are limited indicators of the independence and functional capabilities of an individual [3,4]. To a large extent, ADL records are still manually collected through direct observations by nurses and caregivers in healthcare centers or in homes. Without the support of high-tech products and services, caregivers have to follow their patients during the day and even at night to collect ADL information for behavior assessment, which can be very costly. Hence, attempts to automate ADL recognition and analyze ADL data resolve this dilemma in practice.

The remainder of the paper proceeds as follows: the following section reviews some existing activity recognition projects, and points out the importance of sensor fusion. Section 3 gives a brief review of the *ADL Recognition System*. Section 4 devotes to a time-series sensor fusion model employed in our work. Section 5 briefly presents the system setup and development. Relevant case studies for verifying the proposed model are discussed in Section 6. Section 7 gives the conclusions and future directions of this research.

## 2. Related Work

Digital Health can be described as a convergence of digital and genomic technologies with health, healthcare, living, and society to enhance the efficiency of healthcare delivery [5]. The attention of the researchers in Digital Health has also aroused extensive public concerns. Ermes et al. [6] examined how well the daily activities and sports performed by the subjects in unsupervised settings with wearable sensors can be recognized compared to supervised settings. Tapia et al. [7] presented a system for recognizing activities in the home setting using a set of small and simple state-change sensors. The project HBMS (Human Behavior Monitoring and Support) conducted research on Active and Assisted Living with a focus on supporting elderly people in their daily activities. Their approach is to unobtrusively help a person by reminding her/him of her/his former practice whenever she/he seems to be stuck in an activity [8]. Mo et al. [9] developed an ensemble classifier based on wearable multi-sensors for physical activity pattern recognition, which combined multiple classifiers based on different sensor feature sets to improve the accuracy of physical activity type identification. Zhu et al. [10] used wearable inertial sensors and fiber sensors for ADL monitoring and classification. These sensors attached to different human body parts are used to capture kinetic data. Recognition is achieved by combining neural networks and hidden Markov models. Zheng et al. [11] created a wireless wearable multi-sensor system for locomotion mode recognition, with three inertial measurement units (IMUs) and eight force sensors, measuring both kinematic and dynamic signals of human gait, using a linear discriminant analysis (LDA) classifier. Bayat et al. [12] proposed a smartphone-based activity recognition system, an application of a low-pass filter and a combination of Multilayer Perceptron. The overall accuracy of the classifiers that combined LogitBoost and Support Vector Machine (SVM) was 91.15% when the user held the smartphone in his/her hands. The data was recorded from four volunteers while performing six activities: slow running, fast running, walking, aerobic dance, stairs up and stairs down. Buber et al. [13] developed a recognition system based on tri-axial accelerometer data of a smartphone for the eight activities: walking, running, biking, stairs up, stairs down, sitting, standing and jumping. The simulation was conducted by five volunteers performed those activities with the smartphones in their pockets. The evaluation was performed with two feature selection algorithms (OneRAttributeEval and ReliefFAttributeEval) and six classification algorithms (Bayes net, Naïve Bayes, J48, K-Star, k-Nearest Neighbor (K-NN), and Random Forest) and 10-fold cross-validation. The best recognition rate of 94% was from a combination of 15 features. Feng et al. [14] proposed collecting the data of daily activities solely via a single smartphone, as the mobile devices are becoming increasingly sophisticated and the latest generation of smartphones now incorporate many diverse and powerful sensors. The scope of ADLs was applicable to many normal instrumental ADLs in various comprehensive situations.

Undoubtedly, sensors that underlie the Digital Health applications are very pervasive. Furthermore, more than one kind of sensor should extend the utilization of features. Hence, sensor fusion is the elementary joint to cope with multiple data sources. The sensors used in this work include GPS sensors, audio sensors (i.e., microphones), proximity light sensors, atmospheric pressure sensors (i.e., barometer), direction sensors (i.e., magnetic compasses), and acceleration sensors (i.e., accelerometers), Wi-Fi module, cellular signal module, etc. In order to build a sound theoretical foundation for a practical product, the *ADL recognition system* should achieve both a high-level academic standard and an ultimate satisfaction from real users, so improving the recognition accuracy should be a highly critical task. Ultimately, the time-series based sensor fusion technique is adopted to analyze fast-growing data, which mainly serves as a feedback mechanism of error correction. Pires and Garcia [15] utilized some inertial sensors fusion such as accelerometers, gyroscopes, and magnetometers of the most common wearables for human activity recognition. Furthermore, in [16,17], they proposed several fusion methods based on mobile devices to recognize various daily living activities.

## 3. The ADL Recognition System

To keep track of an elderly person’s activity and present his/her behavior prediction to observers (e.g., doctors, relatives), the *ADL Recorder App* [14] is developed to capture mobile data from multiple embedded sensors, including microphone, Wi-Fi scan module, heading orientation, light proximity, step detector, accelerometer, gyroscope, magnetometer, time stamp, etc. The aim of the *ADL Recorder App* is to collect the phone user’s behavioral context and environmental context where the user resides. This study makes it possible to collect ADL data just by one standard smartphone, which can be accessible to many common users. We also propose the *ADL recognition system* [18] that not only paves a road for recording, detection, recognition, and electronic documentation of user’s ADL but also yields some health-related reports through statistical analysis. Case studies in real-life situations prove the feasibility of our *ADL recognition system*. *ADL recognition server* is hosted on an agent-based Information Management Platform (IMP), which is following the service oriented architecture (SOA)-based publish/subscribe operational mode. IMP largely decouples web-based applications, as a large application can be decomposed into several modules. Different publisher clients can post information to IMP independently, and subscriber clients will benefit from a well-defined interface to access the high-level integrated information. The ultimate goal of IMP is to deliver agility to support any distributed information sharing application. The data flow of this work begins with capturing sensor data from a user’s smartphone, receiving at the IMP server, cleansing and filtering data, *Extracting, Transforming and Loading* (ETL) on the server cluster to the warehouse, running machine learning algorithms on the dataset for analysis, pushing HTTP data module to the presentation layer and providing the dataset to anyone involved. As many users of the *ADL Recorder App* constantly upload data onto the IMP, the data transmission traffic, data storage, and user’s daily activities recognition ends up with a huge burden. Big data frameworks are employed to support the IMP.

Key technologies in the *ADL recognition system* cover audio processing, Wi-Fi indoor positioning, proximity sensing localization, and time-series sensor data fusion. We invent different algorithms for different modules, such as situation-aware computer audition analysis, Wi-Fi-based indoor localization, visible light based localization, and multi-source sensor data fusion. Each module achieves high recognition accuracy. In the end, outcomes from different modules are merged to derive final ADL identification results. Modular programming facilitates the construction of this large system. Each module, such as audio processing, indoor localization, has its independent functionality with an interface to the whole system. During the development, each module experiences interchangeable upgrade gradually. The final ADL results primarily include two elements: the user’s location and the user’s action.

As this work merely relies on the mobile data collected from a single smartphone, and Global Positioning System (GPS) features are not always working for some users, in order to save energy or under indoor environments, we developed an alternative approach to differentiate multiple major locations from analysis on the environmental context, such as Wi-Fi Received Signal Strength Indication (RSSI), Bluetooth and GSM. With the new positions being visited, the number of total Wi-Fi access points increases as new ADL data arrives. This means that the dataset grows both in quantity of records and number of features. Thus, one online learning clustering algorithm is needed to solve these kinds of problems. Moreover, this clustering algorithm is adaptive to distinguish different location-resolutions, such as city, campus, building, apartment, and room levels.

As for the action recognition, various types of machine learning classifiers and data pipelines are applied in each processing module. Bayesian network, hidden Markov model, Gaussian mixture model, random forest, and k-nearest neighbors are used in situation-aware audition module. The J48 decision tree and Bayesian network are applied in a visible light based localization. The online clustering algorithm is invented for global localization. The J48 pruned tree is utilized for final ADL recognition. To manage complex data pipelines of batch jobs, data pipeline management frameworks are recommended such as Luigi (http://luigi.readthedocs.org), Airflow (https://airflow.readthedocs.org), Pinball (https://github.com/pinterest/pinball), etc.

After data fusion and machine learning process in the system, the result of classification is presented as charts, bars, and other visualized forms. On the presentation layer, our work visualizes activities in different formats for multi-purpose end-users who could be a doctor, a data analyst, one of the patient’s family members, or just this patient.

We start to apply time-series based sensor fusion on the fast-growing data. The time-series technique is adopted to analyze motion data. In each motion package, a new data fusion algorithm needs to be applied to sensory data from accelerometer, magnetometer, rotation, orientation, etc. The aim is to discover user’s motion according to the phone’s attitude/status. With the help of motion/transition analysis, hidden ADL patterns can be discovered. Therefore, ADL in each position before and after each transition can be recognized in the way. Furthermore, motion analysis can help recognize ADL through the phone’s various attitudes.

Time-series analysis can serve as a feedback for calibration of the historical ADL results. In step with new ADL types, more new knowledge can be learned and stored in the system. Therefore, as more users continuously provide data, the knowledge database for the unfamiliar environments should grow at the same time. At this moment, we develop a mechanism to utilize the new knowledge to modify the unreasonable results recognized earlier.

## 4. Time-Series Sensor Fusion Model

Harris [19] proposed a waterfall model of hierarchical architecture for data fusion community. Hereby, we enhance this model with time-series features. This model describes the flow of data that operates from the data level to the decision-making level.

Data from sensors are hierarchically categorized into multiple levels. A thermometer on the physical level represents a value in Celsius or Fahrenheit, which is on the data level. Compared to human’s body temperature, the ambient temperature could be roughly regarded as cold, comfortable, hot, or in finer granularity, which is discussed on an information level. One with intent to take some actions to make himself/herself feel better, probably he/she will turn on the heater or air conditioner, which is thought on the decision-making level.

**Figure 1:**
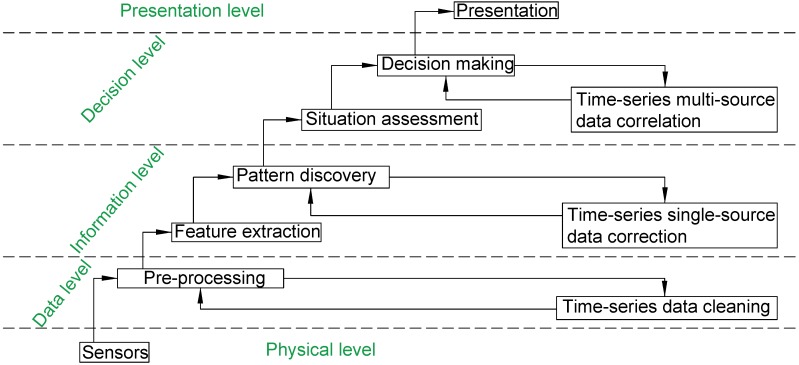
Time-series sensor fusion cascade model with error correction.

Continuously, the entire sensor system is updated with feedback information arriving from the decision-making module. That is to say, the feedback element advises the multi-sensor system on re-calibration, re-configuration, and data gathering aspects. We believe that time-series feedback can be applied not only for the outer loop from the decision-making module to the sensor level but also within each module at every level, as shown in Figure 1:

- At the physical level, multiple sensors keep capturing the behavioral context and environmental context. Physical sensors usually are in “ON” state, the transmission links of data into storage and bottleneck data control are considered.
- When obtaining data from the physical level, the data level checks for physical transmission errors and packages sensing data into “frames”. Time-series filters are responsible for reducing or removing noise, and smoothing data in the pre-processes. The data level also formats the data into packets delivered up to the information level.
- On the information level, data are properly extracted into formatted features. The extraction algorithm for each module varies.
  Wi-Fi-based localization module is built on utilizing the Wi-Fi RSSI feature; acoustic features of environmental sound are extracted and recognized from audio files; activity recognition focuses on the data collected from the accelerometer, gyroscope sensors, etc. Based on the features along the time going, time-series pattern recognition and discovery are conducted.
  A time-series correction feedback can handle error correction on the information level. As an example, due to the unstable Wi-Fi signal, one-round Wi-Fi localization algorithm cannot gain accurate locations. Adding the correction feedback can largely improve the accuracy. In this case, some basic rules are readily applied. For instance, it is impossible for a user to relocate without any motion. Likewise, it is impossible that a phone receives the same Wi-Fi RSSI from every Access Point (AP) after it moves directly forward. Such kinds of impossible rules are by nature useful for corrections.
- on the decision level, through the context assessment, where context awareness is imported, which plays a big role in combining the environmental context and behavioral context. This level is capable of adapting multiple types of features together for high-level decision making furthermore. Time-series correction feedback in this level is responsible for rule-based error correction based on context as well. In practical recognition, it is less likely that one’s ADL pattern is “cooking, sleeping, then cooking” within 30 min. Therefore, an incorrect ADL label of “sleeping” in the middle should be purged accordingly.
- The output of the presentation level might not be as much as that from the decision-making. The presentation is for end-user to review, thus the content should be compact and clear. The output of decision making is usually numerously generated from the algorithm, and such result is more beneficial for the scientists and analysts. Hence, the compact form results need to yield for the end consumers on the presentation level.

### 4.1. Time-Series Data Cleaning on the Data Level

As for the *ADL Recognition System*, one of its aims is to discover a user’s motion according to the phone’s attitude. With the help of motion/transition analysis, ADL patterns can be discovered. In each motion package, the time-series based sensor fusion algorithm needs to be applied to the sensory data from accelerometer, magnetometer, rotation, orientation, etc. Therefore, ADL in each position before and after each transition can be recognized on the fly. Furthermore, motion analysis can help recognize ADL through phone’s various attitudes.

Most earlier work in accelerometer-based activity recognition performed their experiments by placing multiple sensors on several parts of the subjects’ body [20,21,22]. Hip, wrist, arm, ankle were usually selected to attach sensors. Nowadays, Android-based cell phones mostly contain tri-axial accelerometers. The acceleration in three spatial dimensions with the magnetometer sensor and Earth’s gravity can detect the orientation of the device. An accelerometer-based activity recognition system [23] identifies the physical activity a user is performing, such as walking, jogging, climbing stairs, sitting, and standing.

This section is to determine the phone’s attitude and the walking direction by combining multiple types of sensors in addition to accelerometers.

Some practical real-world situations are worthy of taking account. Even holding in one’s palm, it does not really reflect motion direction. It is possible to detect a stride while distinguishing whether the phone is heading in directly the same motion direction or totally the opposite is another challenge. Sometimes, walking backwards also happens.

**Figure 2:**
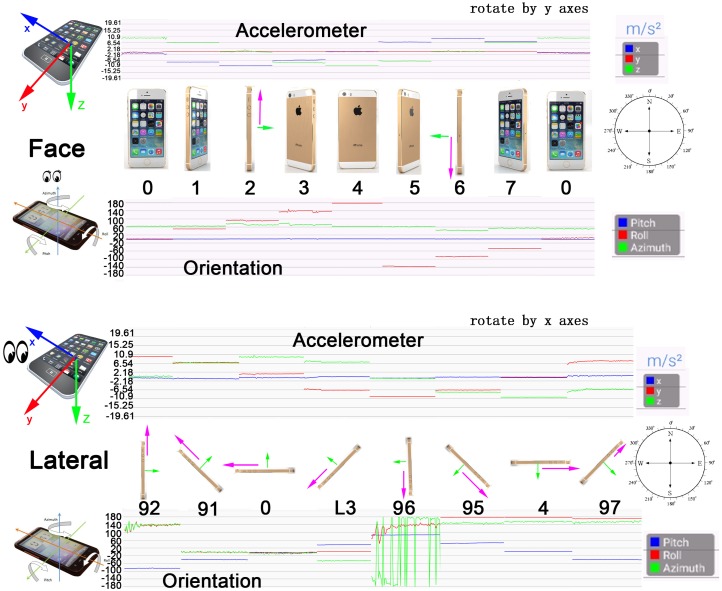
Accelerometer and orientation data (pitch, roll, azimuth) in various attitudes.

Furthermore, detected heading angle is always fluctuating when a smartphone is in the pocket heading downwards to the ground. Obviously seen in Figure 2, the detected raw data are very different from the correct walking direction in case:96. Thereafter, a time-series filter is needed, such as moving average filter and Exponentially Weighted Moving Average (EWMA) [24]. Smoothing heading direction for case:96 in Figure 2 is one example applied here.

In addition, time-series recognitions over several intervals with feedback are necessary. By case study, some basic anchor points are observed:

- It is most likely to gain a precise heading direction when a phone is in the horizontal viewing attitude (case: 0 in Figure 2).
- It is possible to determine the orientation in a pocket by Kalman filter with current detected attitudes and how the phone is turning for viewing.

### 4.2. Time-Series Single-Source Data Correction on the Information Level

Primarily, errors on the information level result from two sources: improper recognition approach and defective data passing from data level. Different pedometer Apps have a different method to count steps and calculate calories, yielding almost different values [25]. Unfortunately, all of those values can not be correct at the same time, thus causing errors.

Errors on the data level can also affect the recognition results on the information level. Usually, vulnerable Wi-Fi signal strength from a certain AP varies from time to time. Apparently, the strength of the magnetic field may vary a lot around some electric appliance, such as a sound stereo receiver, a refrigerator, a color TV, etc. Even the magnetic field of door access control systems can interfere with the orientation. These noises interfere immediately and affect the recognition results on the information level.

### 4.3. Time-Series Multi-Source Data Correlation on the Decision Level

**Figure 3:**
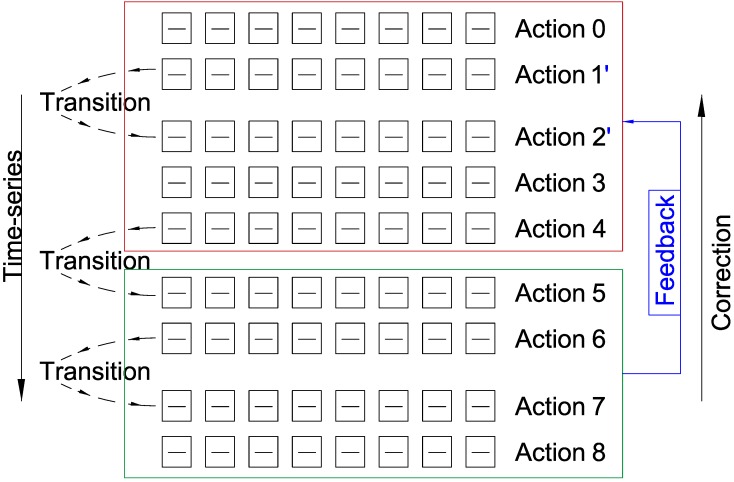
Error correction framework of time-series based sensor fusion.

Time-series analysis can serve as a feedback for calibration of the prior ADL results. In step with new ADL types, new knowledge is increasingly learned by the system. Then, we will develop a mechanism that utilizes the new knowledge to modify the unreasonable results recognized earlier on the decision level, illustrated in Figure 3.

## 5. System Development

Specialization in everything from front-end to back-end is involved in this full-stack development.

The *ADL Recorder App* offers a native version for Android and iOS platform. It requires Java language for Android App development and Swift language for iOS App development. The agent-based Information Management Platform (IMP) is first developed in Java-based server on the Amazon Web Services (AWS), and the Scala language takes part in several new features. For the *ADL Recognition System*, Apache Kafka is a better choice for its high performance and compatibility with a distributed system.

Deployment of Kafka can ensure that each record file will not get lost in high throughput; hereafter, Kafka copes with the stream-processing to receive numerous data packages.

ETL serves to load processed data into a data warehouse, transform as the input and load to the MySql/MongoDB database. Representational state transfer (RESTful) web services provide interoperability between the AWS and publish/subscribe clients.

Feature extraction and processing of audio, Wi-Fi localization and other raw data are conducted by Python scientific language in the proof-of-concept stage. In the production stage, the computation work in the server is quite heavy due to the massive data volume.

The amount of ADL data on the IMP can increase day and night, and the difficulty and complexity of discovering and analyzing ADL patterns are becoming much more serious, so the conventional stand-alone smartphone-based human activity recognition is not always efficient. For adapting to the big data era, we introduce a big data distributed computing platform (such as Spark and Hadoop) in the system so that we can solve some critical issues related to distributions like latency and network communication for improved performance. The requirement is decomposed into two groups: batch computation and streaming computation. Batch computation is to learn/train historical ADL data; streaming computation is to provide the latest ADL result on the fly. Spark processes data in-memory while MapReduce pushes the data back to the disk after processing it. The principle of the Spark engine is to analyze the codes and optimize the flow of the data processing before actually running the codes. Therefore, Spark outperforms Hadoop in terms of efficiency [26]. Streaming computation is processing on a streaming data and returning a streaming result. Storm, Spark Streaming, S4, and Heron are some popular streaming computation engines. Spark Streaming and Spark are similar in principle. Both of them are developed in Scala, a programming language friendly to data scientists. Accordingly, we apply Hadoop for the batch computation and Spark for the streaming computation.

In-memory databases can be applied to speed up the *ADL Recognition System*. Redis, which is a popular in-memory database known as a remote dictionary server, has been proved to outperform others such as Cassandra, HBase, MongoDB, and OrientDB regard overall performance [27]. When dealing with time-series analysis and machine learning driven predictions, a Spark framework shows an accelerated performance after being combined with a Redis database. Shoolman (https://www.infoworld.com/article/3045083/analytics/give-spark-a-45x-speed-boost-with-redis.html) has shown that, after combining Spark and Redis, the efficiency of time series data processing is 45 times faster than using Spark alone. Redis is generally applied at the end of a business logic pipeline after Spark training: it saves time to train model, which are delivered by Spark, and make predictions. The final predicted results are passed to customers by Redis via its Publish/Subscribe messaging mechanism.

Luigi is a batch jobs management framework written in Python, which can be easily integrated with service architectures built with Spark or Hadoop. It helps to monitor and alarm errors when batch jobs are executing. Luigi has a more extensive support community, and it has more maturity compared with Airflow and Pinball. With the assistance of Luigi, batch jobs can easily resume at the broken steps without running from the beginning. This allows developers to focus more on ADL business logic.

Apache Mahout is a machine learning engine which is developed specifically for running machine learning algorithm in Hadoop Distributed File System (HDFS). Another frequently-used machine learning engine, Spark MLlib, is growing in the big data ecosystem on a distributed system. Spark MLlib is high speed in running complex machine learning algorithms.

Moreover, Spark MLlib have interfaces for common machine learning algorithms, including classification, regression, decision tree, clustering, etc.

C# language with .NET MVC 5 are selected to publish ADL results for *ADL Reviewer App*, which is used by subscribing customers. A bunch of JavaScript frameworks are employed for the representation website, such as AngularJS, JQuery, Telerik Kendo UI, AmCharts, Bootstrap, etc. For A/B test, we develop another ADL reviewing web application using the Play Framework, together with Spark engine, and the Scala programming language is utilized from data analysis to web development.

### Optimization of Battery and Network Traffic

The optimization problems on the client side consist of minimizing battery consumption and reducing data package transmission. By default, the *ADL Recorder App* captures sensor data every 3 min. Although it meets the requirement in this study, it can not fit for highly real-time needs—for instance, fall detection. Thus, we face a trade-off between performance and efficiency.

The sampling rate of sensors in the Android system is programmable, but a high frequency largely drains the battery. The default data delay is suitable for monitoring typical screen orientation changes and uses a delay of 200,000 microseconds. You can specify other data delays, such as SENSOR_DELAY_GAME (20,000 microsecond delay), SENSOR_DELAY_UI (60,000 microsecond delay), or SENSOR_DELAY_FASTEST (0 microsecond delay) (https://developer.android.com/guide/topics/sensors/sensors_overview). The sampling rate of each kind of sensor in the iOS system varies. For example, 100 Hz is for BLE, and 50 Hz is for gyroscope, accelerometer and other Inertial Measurement Units (IMU).

The changing rate of an average human’s movement is relatively smooth. Based on this, the *ADL Recorder App* captures each piece of sensory data under a low sampling rate of 5 Hz. As for each sensor type, the differences between each subsequent sample is checked. The app only captures a data package and caches it onto device memory only if one of the difference amounts across multiple sensors is larger than a pre-set threshold. That is to say, if none of the sensors changes beyond its threshold, the App will not generate a data package. It leads to low costs when the smartphone is still outside on a dark night. The *ADL Recorder App* can be configured to upload data packages on the fly or only in a Wi-Fi environment in order for it to save a lot of data costs, networking traffic and battery consumption. By default, it lasts for 15 s in each data capturing session, and it sleeps for three-minute intervals before another new session. The data package size of each session is around 25 KB. Supposing that the smartphone is actively used for 18 h a day, the total disk consumed will be 18 h × 20 times/hour × 25 KB = 9 MB daily per device. If the microphone module is disabled, the data package size of each session is less than 2 KB, so each device generates 18 h × 20 times/hour × 2 KB = 720 KB daily.

## 6. Case Study and Discussion

Basically, our work had two development stages. In stage I, we conducted an internal acceptance testing for the proof-of-concept. The users needed to perform a think-aloud feedback, which was provided as an action self-report page in the App. Our subject users included the researchers, developers, and ones who had interest in this project. More than 30 volunteers participated in this testing stage. Much data analysis was done on collected raw data, for each separate module and particularly focused on validation of the machine learning classifier. In stage II, our system was released to the open public. Based on the knowledge learned in the previous stage and onwards, the ADL recognition service was enhanced. Meanwhile, we developed a web application to collect customers’ valuable feedback. Along with the increasing knowledge, the *ADL Recognition System* keeps being upgraded to deliver increasingly validated values.

### 6.1. Case Study A in Stage I

During the first proof-of-concept stage, a research version App and a recognition server were implemented and underwent Alpha testing. Ten researchers and participants readily contributed the very first testing data. The *ADL Recorder App* was installed in everybody’s smartphone, and those participants lived with their smartphone all day for two months. Each of them needs to fill in a UI form including the position and actions at that moment on his/her smartphone, similar to a think-aloud process, providing the ground truth. Each recording was launched manually only until the ADL form was filled.

The subjects used the *ADL Recorder App* mostly in their apartment and their friends’ houses. They were living in multiple states, including Iowa, Illinois, Wisconsin, Florida, Michigan, New York, and California. The ADL types under the study contain having meals (breakfast, lunch, dinner), cooking in a kitchen, chopping vegetables on a cutting board, washing dishes, using a bathroom, flushing toilet, washing in a bathroom, walking in a living room, working on a desktop in a study room, sitting on a couch, browsing on a smartphone, and so on.

Multiple recognition modules work separately. The Wi-Fi-based indoor location attempted to localize the subject; and the sound classification module was applied to predict action and also to retrieve location information. For example, hearing the sound of running water suggests that this ADL was close to a faucet either in a kitchen or inside a bathroom. Various sensors were adopted to substantiate a prediction, such as orientation, microphone and GPS module. The time at the current moment was equally stellar.

The recognition accuracies of four of them who collected the most data were 92.35%, 96.15%, 98.92%, and 99.17% [14].

### 6.2. Case Study B in Stage I

Our proposed approach was verified through a full-scale case study that took place in National Central University. Ten graduate students participated in the study. For this case study session, the research version App was used. The recording session was manually launched at will. Our *ADL Recognition System* observed that they were living on campus, staying in some classrooms, going shopping in some supermarkets, taking trains, living in their parents’ city, etc. Compared with the ground truth on the UI forms, the recognition accuracies of them were all above 90% [14].

### 6.3. Case Study C in Stage I

In this case study phrase, a product version App was tested by a limited number of end-users to obtain feedback on the product quality. Our objective is to discover users’ life patterns. The same as before, the participants were living in real environments. The *ADL Recorder App* was quietly running on their smartphones 24/7, and it captured a data package every three minutes by default. The subjects’ ADL types were not confined anymore. The end users would stay at home, drive on the road, work in an office, join a seminar at school, do shopping in a grocery store, take a flight, stay in an airport, etc. The *ADL Recognition System* processed each user’s data, based on the ADL domain knowledge, and it profiled their activities during those periods of time. In this case study, because the activities took place anywhere, the Global Wi-Fi Positioning algorithm [28] was applied for localization, and high-level ADL results were produced accordingly, such as walking at the Narita International Airport in Tokyo Japan, sitting in a conference room at Wanda Reign Hotel in Wuhan China, running in the fitness center at Iowa State University, Ames, IA, USA, having dinner in a cafeteria at National Central University, etc. The principle of the Global Wi-Fi Positioning algorithm is to fully take advantage of the information retrieved from access points, including RSSI, and the Service Set IDentifier (SSID). The algorithm was evaluated across several remarkably different situations, including localizing a room in a complex building, localizing a building on campus and localizing in some public facilities. Once necessary knowledge is collected and learned, localizations across these kinds of situations can be obtained. In this case study phrase, time-series data cleaning on the data level, single-source data correction on the information level and multi-source data correlation on the decision level are broadly employed. They significantly exert positive effects on many aspects, such as modifying missing raw sensory data, error correcting potential classification errors for each module, and reducing unreasonable predictions in temporal ADL sequences.

Evaluation of battery consumption was conducted by observing ten users’ smartphones. The battery consumption of this App was only around 9% of all underlying processes, despite a heavy usage of multiple sensors in the *ADL Recorder App*. The reason is that many sensors keep working all the time to support many Apps on smartphones. The battery consumption of the *ADL Recorder App* can reduce to 2–4% if the audio module is off, as only Wi-Fi related processes are largely energy-draining, such as uploading data through networking and scanning APs.

Audio information retrieval and visible light-based indoor localization are two essential techniques in the *ADL Recognition System*. We proposed our algorithms that were suitable for our use cases. Audio information Sound can detect fine-granularity ADLs, for example, according to the environmental sound and event sound, it concludes someone’s position and action. Derived from the audio information, common sense makes it possible to tell that someone was stirring eggs and cooking in the kitchen, and later he was vacuuming the carpet.

The characteristics of visible indoor light represent much abundant information. Here is one example, and the received light strength is evidence of predicting a relative distance from a luminary. It is able to yield a precise location within a small area in a very distinguishable feature. A light-based localization system is proposed in this work, which aims to estimate smartphone users’ locations and ADLs via machine learning algorithms. Evaluations in both the experimental environment and real-world environment have been conducted and indicated that decimeter indoor location accuracy can be achieved.

Note that, for each subject or customer, the most valuable service is not covering all of their possible ADLs happening on the whole day. It should focus on the significant ADLs or the ADLs worth their attention. For instance, for an elderly woman, the recognition of coughing is much more important than having conversations; for a kid, the recognition of crying and feeding are far more important than playing. Therefore, we always try our best to provide a personalized service and focus on substantial ADLs. By a series of comprehensive case studies, we have corroborated that the *ADL Recognition System* is capable of ADL recognition and localization with high accuracy.

### 6.4. Some Thoughts in Stage II

Stage II was similar to a Beta testing. The product version App collected end-users’ data continuously days and nights. To protect users’ privacy, the microphone feature could be disabled, thus no information was retrieved from the audio module.

The primary objective in this stage was to display a user’s historical ADL on web pages, and furthermore to generate daily/weekly/monthly ADL summaries. Bar charts, histograms, and pie charts were created to represent various ADL trends. The multi-source data correlation on the decision level was introduced to ensure the correctness. Meanwhile, the system is always evolving based on the users’ firsthand experience.

More examples are available on the page http://web.cs.iastate.edu/ ~yunfei/adl/.

## 7. Conclusions and Future Works

Indeed, ADL recognition has evoked notable scientific interest in the past decade, primarily because ADL is a good indicator to measure one’s daily living ability—for example, eating, bathing, getting dressed, toileting, transferring and continence. Being able to perform ADLs while aging is directly linked to independent living, as physicians and adult care social workers can use ADLs to determine if a person needs assisted living or should be placed in a nursing home, as well as the way in which individuals respond to such difficulties.

The ubiquitous sensor applications and the rapid development of data processing techniques, such as big data and machine learning, make it possible to build an automated ADL recognition system. In this work, we proposed a comprehensive solution to ADL recognition and hidden pattern discovery. The *ADL Recorder App* is a client application running on a user’s smartphone. It collects data from multiple embedded sensors, including the microphone, Wi-Fi scan module, heading orientation of the device, light proximity, step detector, accelerometer, gyroscope, magnetometer, etc.

The back-end application, *ADL Recognition System*, is hosted on an Information Management Platform (IMP). The *ADL Recognition System* keeps processing the coming data package from numerous clients in batch and on the fly. Various machine learning algorithms account for each type of sensor and the combination of sensors. The time-series sensor fusion technique underlies the processing for each module, including indoor localization, activity recognition, audio processing, and other substantial factors. Afterwards, the final ADL results will be presented in various visualization types to multi-purpose data consumers.

Since smartphones become popular devices with which users are carrying every day, they do not need to buy additional devices for the system. Compared with different kinds of traditional methods, the *ADL Recognition System* gives users a better experience in cost and comfort. Case studies have verified that the proposed system for an accurate ADL recognition and life pattern discovery. Moreover, dataset and related APIs for new researchers and programmers are readily provided.

There are still several directions for future work. Higher recognition accuracy on each processing module and the overall final ADL results are always in need. Recognition of more ADL types will be explored for customized users. It makes storage and processing costs exceptionally high for long-term maintenance, hence data compression and summarization at least some old data will be considered.
